# Vertical Signalling Involves Transmission of Hox Information from Gastrula Mesoderm to Neurectoderm

**DOI:** 10.1371/journal.pone.0115208

**Published:** 2014-12-16

**Authors:** Nabila Bardine, Gerda Lamers, Stephan Wacker, Cornelia Donow, Walter Knoechel, Antony Durston

**Affiliations:** 1 Leiden University, Institute of Biology, Sylvius Laboratory, Leiden, The Netherlands; 2 University of Ulm, Institute of Biochemistry, Ulm, Germany; Instituto Gulbenkian de Ciência, Portugal

## Abstract

Development and patterning of neural tissue in the vertebrate embryo involves a set of molecules and processes whose relationships are not fully understood. Classical embryology revealed a remarkable phenomenon known as vertical signalling, a gastrulation stage mechanism that copies anterior-posterior positional information from mesoderm to prospective neural tissue. Vertical signalling mediates unambiguous copying of complex information from one tissue layer to another. In this study, we report an investigation of this process in recombinates of mesoderm and ectoderm from gastrulae of *Xenopus laevis*. Our results show that copying of positional information involves non cell autonomous autoregulation of particular *Hox* genes whose expression is copied from mesoderm to neurectoderm in the gastrula. Furthermore, this information sharing mechanism involves unconventional translocation of the homeoproteins themselves. This conserved primitive mechanism has been known for three decades but has only recently been put into any developmental context. It provides a simple, robust way to pattern the neurectoderm using the *Hox* pattern already present in the mesoderm during gastrulation. We suggest that this mechanism was selected during evolution to enable unambiguous copying of rather complex information from cell to cell and that it is a key part of the original ancestral mechanism mediating axial patterning by the highly conserved *Hox* genes.

## Introduction

Determination of regional specificity along the anterior-posterior (A-P) axis of the vertebrate *Xenopus laevis* and of all other vertebrates begins during gastrulation. This patterning involves interactions between the Spemann organizer (SO) and surrounding tissues that are key events leading to genesis of the basic body plan [1]. Classical embryology also revealed a remarkable phenomenon known as vertical signalling, a mechanism that copies A-P positional information from non-organiser mesoderm (NOM) to overlying neurectoderm during gastrulation [2]. Nieuwkoop showed that the A-P pattern of the amphibian embryo is generated in the developing nervous system (neurectoderm) during gastrulation by two types of signals: activation and transformation [3]. These signals are emitted by mesoderm and act on ectoderm. The SO secretes activation signals that induce neuralisation of the ectoderm but induce only an anterior neural identity (presumptive forebrain) [4]. Tissue recombination and grafting experiments indicated that this patterning occurs similarly in an amniote (the chick embryo) as in the anamniote Amphibia [5] and also that transformation signals originate from NOM (lateral and paraxial mesoderm) and that they induce a progressively more caudal identity of the neural tissue [5]–[11]. Known signalling pathways have been proposed to be involved in transformation: these include retinoids [11]–[13], *FGFs* [14] and *Wnts* [15]. Elevated concentrations of these signalling molecules cause posteriorisation by inducing relatively posterior positional values in the neurectoderm and each of these factors has been proposed to act as a posterior to anterior gradient within the embryo [8]–[13]. These factors possibly mediate some of the known planar signals that act along the A-P axis of the germ layers during gastrulation. However, planar signals do not fully account for the properties of neural transformation. The use of exogastrulae and other approaches revealed a second type of signal. It appeared that posterior neural markers were expressed only at the border between ectoderm and mesoderm in exogastrulae, excluding the existence of very extensive planar signalling [16]. These and other observations rule out that axial patterning is fully accounted for by planar signals and the evidence actually indicates that the second type of signalling: vertical signalling is the more important in generating the A-P pattern of the neural plate [16]–[19]. The nature of the molecules involved in vertical signalling remains unclear.

We reported previously that expression of the A-P determining *Hox* genes begins during gastrulation in *Xenopus laevis*. This initial expression starts in NOM tissue in the mid-gastrula [20]. It presumably corresponds with the initial “*Hox* induction field” or “opening zone” that has been reported in other vertebrates [21], [22]. This is the place where *Hox* codes are first available and later, when these mesodermal cells involute and come to lie underneath prospective neural tissue, the same A-P information spreads to that neural tissue by the end of gastrulation [20]. Interestingly, use of a tissue recombination assay, the wrap assay showed a requirement for NOM and SO to induce *Hox* gene expression in the neurectoderm [23]. The SO induces the embryonic ectoderm to a neurectodermal identity and the NOM induces expression of various *Hox* genes in the neuralised ectoderm ([23], reviewed in [24]). These steps appear to correspond to Nieuwkoop’s activation and transformation steps, respectively. In this study, we investigated the importance of the *Hox* genes for vertical signalling using the wrap assay to provide a controlled setting for investigating signalling from mesoderm to neurectoderm. We found that *Hox* expressing NOM mesoderm provides positional information for the adjacent neurectoderm. Moreover, in these wrap assays, mesodermal expression of each of the *Hox* genes that we investigated appears to be necessary as well as sufficient for inducing the expression of the same gene in the neurectoderm. The absolute requirement for mesodermal expression of the homologous *Hox* gene is striking. It indicates an extraordinarily high degree of specificity for vertical signalling: a feature that was already anticipated from the embryological data [2]. Using recombinant *Hox* proteins, we also detected transfer of the homologous *Hox* proteins between NOM mesoderm and neurectoderm during this non cell autonomous *Hox* autoregulation. We suggest that this is the basis of the extraordinary specificity of this mechanism. We also detected *Xenopus Hox* protein uptake from the medium by *Xenopus* embryonic cells as well as Drosophila imaginal discs. Considering that *Hox* codes are thought to be synonymous with A-P positional information and that the NOM induces neurectodermal *Hox* gene expression, our data suggest that expression of individual *Hox* genes is copied specifically from NOM to neurectoderm during neural transformation. This information sharing transfer involves a peculiar protein transfer of the homeoproteins themselves as has previously been described by others [25]. This primitive mechanism has presumably been conserved in evolution to enable specific information sharing between tissue layers in a very simple and direct manner.

## Results

### Hox expression in the mesoderm is necessary and sufficient to induce neurectodermal Hox expression

**Figure 1:**
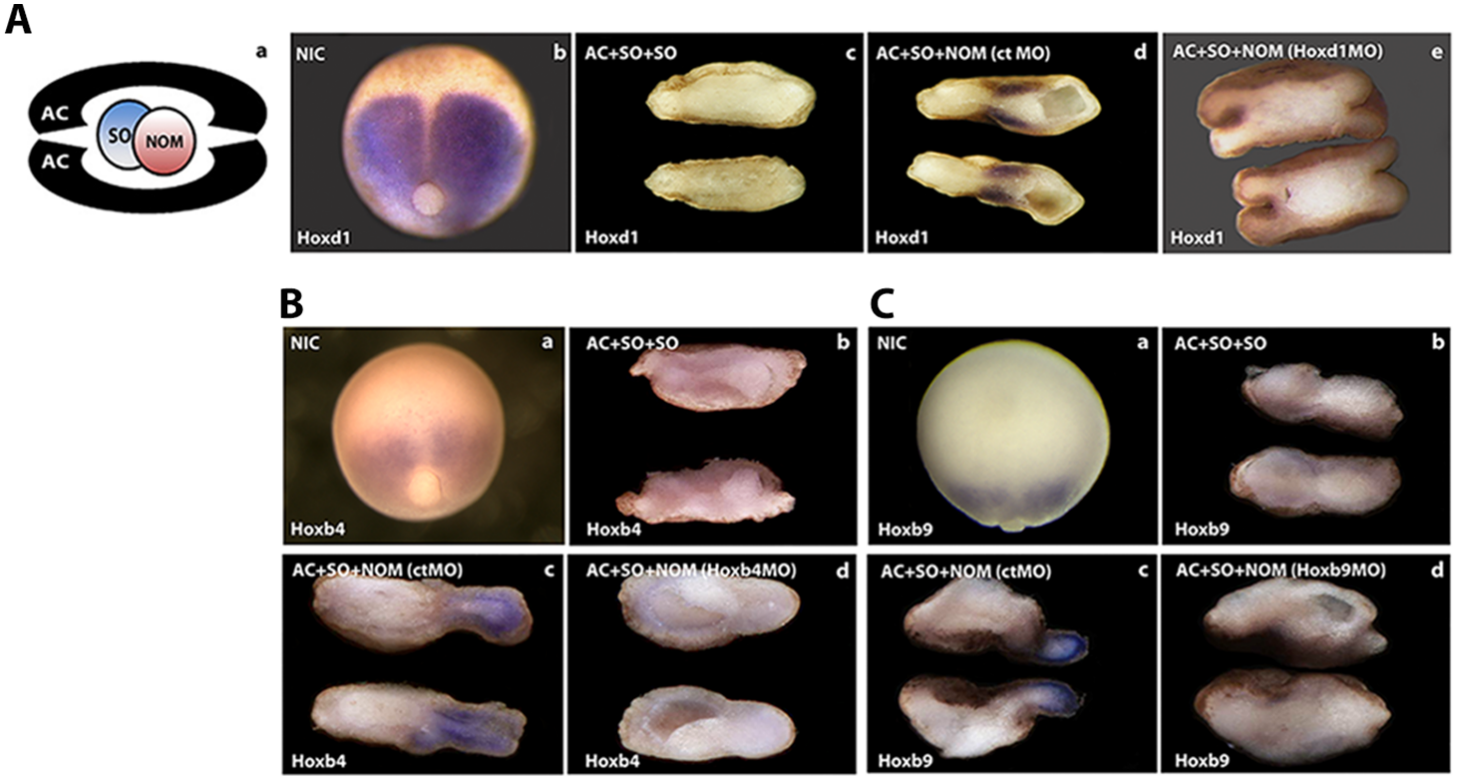
Mesodermal *Hox* loss of function of a single *Hox* gene prevents neurectodermal *Hox* expression of the same *Hox* gene. **Aa**, Wrap assay consists of a piece of non-organiser mesoderm (NOM) and a piece of Spemann organiser mesoderm (SO) combined between two ectodermal animal caps. All tissues are excised from early gastrulae (st. 10a) **Ab**, **Ba**, **Ca:** External views of late gastrula stage *Xenopus laevis* expressing *Hoxd1, Hoxb4* and *Hoxb9* respectively. Note that the midline of the embryo, overlying the SO, does not express any *Hox* gene. **Ac**, **Bb**, **Cb:** wraps containing only SO explants [AC(SO/SO)AC]. **Ad**, **Bc**, **Cc:** wraps containing SO and NOM treated with control morpholino (ctMO) [AC(SO/NOM+ctMO)AC]. **Ae**, **Bd**, **Cd**, wrap with NOM treated with *Hoxd1-, Hoxb4* and *Hoxb9* MO’s respectively. Please note that in each case, only the wraps containing NOM and SO show *Hox* expression in the neurectoderm (**Ad**, **Bc**, **Cc**) and those containing only SO do not show any expression in accordance with the embryo’s lack of *Hox* expression in SO (**Ab**, **Ba**, **Ca**, and **Ac**, **Bb**, **Cb**). In each case, *Hox* MO treatment of NOM mesoderm also prevents the expression of the homologous *Hox* gene in neurectoderm (**Ae**, **Bd**, **Cd**). These wraps were fixed and analysed 6–8 hrs after they were made. Each photo of two recombinates or an embryo in this figure is representative of at least 20 recombinates or embryos, all showing the same result.

**Figure 2:**
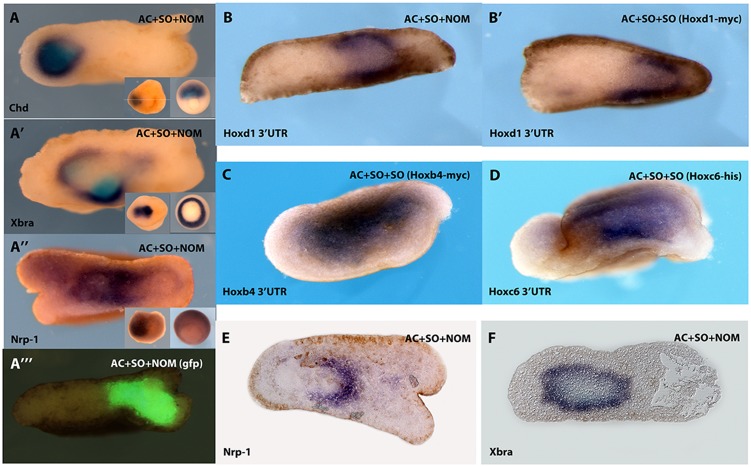
Mesodermal ectopic expression of a single *Ho*x gene copies expression of the same *Hox* gene from mesoderm to neurectoderm. **A– A’’’, E, F** Localisation of mesoderm and neurectoderm in the wrap assay shown by expression of the mesodermal markers *Chordin (Chd,* A*)* and *Brachyury (Bra,* **A’** *)*, and the neural marker *Nrp1* (**A”**). In **A’’’**, lineage labelling by ectopic expression of GFP in the NOM only. These recombinants were analyzed 6 to 8 h after tissue healing. These data show that there is no tissue intermingling during wrap culture. Mesodermal *Chd* (expressed in SO, **A**) and GFP (within NOM, **A’’’**) do not mix with each other within the time course of wrap culture. Consistently, *Bra*, a pan mesodermal marker, is expressed in both types of mesoderm in accordance with its expression domain in the embryo (**A’**).The neural marker *Nrp-1* is expressed in the space between mesoderm and the outermost ectodermal layer of the wrap, consistent with its known pattern of expression within the embryo (**A’’**). **B–B’**: Induction of *Hoxd1* **B:** A wrap containing SO and NOM and ectoderm [AC(SO/NOM)AC] shows induction of *Hoxd1* in the neurectoderm as well as mesoderm. **B’, A** wrap containing normal SO and SO ectopically expressing *Hoxd1* also shows the induction of endogenous *Hoxd1* in the neurectoderm as well as in the mesoderm. Endogenous Hoxd1 expression was detected using a 3′UTR probe that recognizes only the endogenous messenger. **C**, ectopic *Hoxb4* in SO induces its own expression within the neurectoderm and in the mesoderm as in **B’. D**, wrap as in **B’** and **C** but with ectopic *Hoxc6* expression. This shows induction of *Hoxc6* in neurectoderm and in the mesoderm. We used 3′UTR probes to detect expression of the endogenous mRNA’s in each of these experiments. **E, F** sections showing expression of *Nrp1* (neural) and *Bra* (mesodermal) in control or standard [AC(SO/NOM)AC] recombinant. **E** *Nrp1* expression is internal in the recombinant but excluded from an internal cell mass that is clearly the mesoderm. It is particularly strong around one end of the cell mass which is the neural inducing SO. Expression is also absent from the very outer layer of the recombinant, which represents the outer non neural layer of the neurectoderm. **F:** *Bra* expression is in an internal cell mass (the mesoderm). Please note that the germ layer markers *Bra, Ch*, and the mesodermal lineage label GFP are confined to an internal cell mass excluding tissue intermingling and that Hox expression is detected in neurectoderm as well as the mesodermal cell mass. Each photo in this figure represents at least 20 recombinants and embryos with consistently the same results.

We have shown previously that zygotic *Hox* gene expression is first initiated in the non-organizer mesoderm (NOM) in the *Xenopus* mid gastrula (St. 10.5) [20]. This expression then spreads during gastrulation to the overlying prospective neurectoderm that was induced from embryonic ectoderm by signals from the Spemann organizer (SO). We investigated whether *Hox* expression in the gastrula’s NOM causes *Hox* expression in the neurectoderm. This was done using a recombinant wrap assay, in which pieces of SO and NOM mesoderm were combined with two animal caps (Fig. 1A). The main advantage of this assay is that it mimics the embryonic situation with respect to proximity and physical connectivity of SO, NOM and neural tissue, but still allows the independent manipulation of different parts of the early embryonic tissues by loss and gain of function techniques. It also offers the advantage of clear consistent tissue separation [20], [23] (Fig. 1A). Interestingly, a combination of two SO explants within a single wrap is in itself not sufficient to induce *Hox* gene expression, despite causing neuralization of the animal cap (Fig. 1Ac, 1Bb, 1Cb). These results show that this assay mimicks the embryonic situation (Fig. 1A, B, C) and they demonstrate a requirement for a NOM derived signal for induction of *Hox* expression in the clearly neuralised animal cap. We used loss of function via morpholino anti-sense nucleotides to investigate whether a single *Hox* gene knockdown in NOM affects neurectodermal expression of the same gene. MO’s for each of *Hoxd1*, *Hoxb4* and *Hoxb9* respectively were injected into zygotes and the ability of NOM from the injected embryos to induce neurectodermal expression of these *Hox* genes in wraps was examined in comparison to NOM loaded with control MO (ctMO). Fig. 1 shows that NOM knockdown of each of these *Hox* genes prevented expression of the same gene in the neurectoderm (Fig. 1Ae, Bd, Cd) whereas ctMO loaded NOM did not impair neurectodermal *Hox* expression in wraps (Fig. 1Ad, Bc, Cc). These results indicate a specific requirement for expression of a particular *Hox* gene in NOM for its own expression in overlying neuralized ectoderm. Clearly, no other Hox gene or developmental regulator coexpressed in wraps can substitute this requirement, although there are obviously other routes to inducing neurectodermal *Hox* genes *in vivo*. Conversely we asked whether ectopic expression of a single *Hox* gene in mesoderm can induce its own expression in the neurectoderm. In this gain of function approach we took SO grafts from embryos zygotically injected with a single *Hox* mRNA. An explant of SO expressing this single *Hox* gene was then combined in a wrap with a wild type SO graft and 2 animal caps. The untreated SO was included to exclude that *Hox* ectopic expression blocks a necessary SO function. When *Hoxd1*, *Hoxb4* or *Hoxc6* respectively were ectopically expressed in SO, each efficiently induced its own expression in neurectoderm in such a wrap recombinant (Fig. 2B’, C and D). We detected induced expression of the endogenous *Hox* gene using a 3′ UTR probe that does not recognize the ectopically expressed messenger. These results show that ectopic expression of a single *Hox* gene in SO induces expression of the same gene in neurectoderm.

We also show in Fig. 2 that these wrap explants show the normal expression of tissue layer markers as seen in a normal gastrula (Fig. 2A, A’, A”, A”’, E and F). This combinatorial tissue assay shows no intermingling between the different mesoderm types or between mesoderm and ectoderm during wrap culture and the markers and lineage labelling clearly show different localisations of mesoderm and neurectoderm within the wrap (See Fig. 2 legend for further description). There is clear correspondence of Hox expression with expression of both mesodermal and neurectodermal markers.

Altogether, our results show that in the wrap setting, a single *Hox* gene expressed in the mesoderm is necessary and sufficient to induce its own expression in overlying neurectoderm.

### Vertical signalling and Hox protein transfer

**Figure 3:**
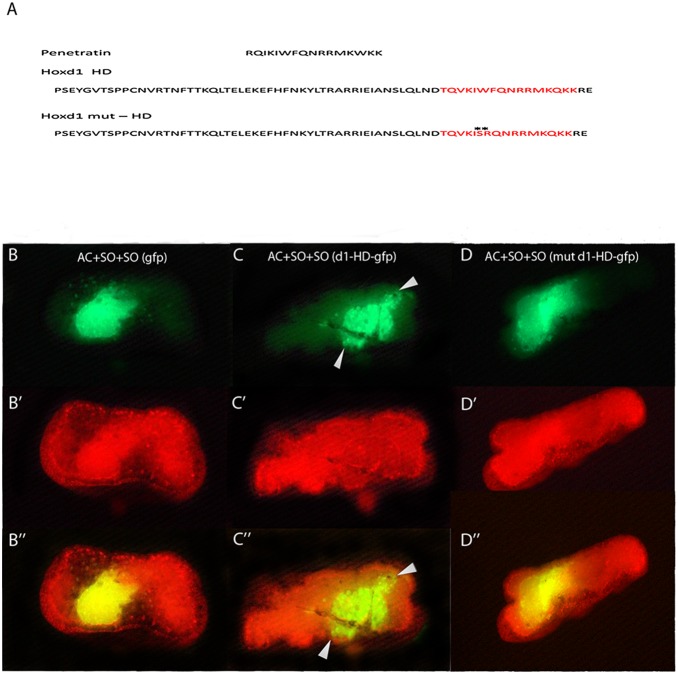
*Hoxd1* homeoprotein containing a penetratin sequence is transferred from mesoderm to neurectoderm and its homeodomain plays a cargo function for *gfp*. **A:** Penetratin sequence is shown. Above, *Hoxd1* homeodomain (HD) contains a penetratin like sequence (in red). This conserved sequence is a feature of all homeodoproteins. Below, two amino-acids WF within the penetratin were mutated into SR (highlighted by stars) to create a mutated *Hoxd1 HD, mut HD*. This mutation abrogates the transfer function of the HD. **B–D”:** Localisation of different fluorescent chimeric GFP proteins in recombinants after 6–8 hrs of culture. These proteins were introduced into wrap recombinates in SO. **B:** wild type GFP. **C:** wild type GFP coupled to wild type *Hoxd1* homeodomain (*d1-HD-gfp*). D: the mutated homeodomain version coupled to wild type GFP (*mut d1-HD-gfp*).The signal has spread in *d1-HD-gfp* but not in wild type GFP or *mut d1-HD-gfp.* The GFP fluorescence is combined with phalloidin staining to increase its visibility. **B, B’, B”:** GFP expressed in the SO stays confined within the SO explant and fails to spread into surrounding neurectoderm. **C, C’, C”:** *d1-HD-gfp* spreads outside the mesodermal explant to the neurectoderm (spreading indicated by arrowheads). **D, D’, D”:** *mut d1-HD-gfp* protein shows a SO localisation pattern as shown by wild type GFP. Each photo in this figure is representative of 23 recombinates, each of which gave the same result. Homeoproteins contain a separate HD sequence regulating homeoprotein secretion as well as the penetratin sequence. The mutations we made were in the ‘penetratin’ uptake regulating sequence. Please note that d1-HD-GFP, mut d1-HD-GFP and GFP in Fig. 3 evidently diffuse less than Myc tagged Hoxd1 in S1 Figure. This is expected, due to the large size of GFP.

Tissue recombination as in the wraps used in this study and in grafting experiments in various vertebrates have shown consistently that mesodermal signals induce the neural A-P pattern (see introduction, [3], [5], [6]). Major signalling pathways, including *Wnts*, Retinoids and *Fgfs* have been suspected to have a role [11]–[15]. Another type of molecule that may be involved in vertical signalling is the *Hox* proteins themselves. This idea was inspired by results from Prochiantz and colleagues who showed that homeoproteins move from cell to cell due to the existence of a special sequence, penetratin, within the homeodomain [25]–[27]. This led us to investigate whether *Xenopus Hoxd1* protein possesses such a sequence. Fig. 3A shows that *Xenopus laevis Hoxd1* protein has a penetratin sequence in its homeodomain. We constructed a labelled (*myc* tagged) *Hoxd1* and analysed its ability to translocate from mesoderm to neurectoderm. *Myc-Hoxd1* was ectopically expressed in SO as in S1 Figure. The labelled (*myc* tagged) *Hoxd1* protein was seen to translocate from the labelled SO mesoderm to unlabelled mesoderm and neurectoderm tissues in these wrap recombinates. *Hox* proteins translocate due to their penetratin sequence, which is known to confer transfer capacity and cargo functions that depend on key amino-acids in the penetratin sequence (see Fig. 3A). Mutation of these amino acids abolishes the protein’s ability to translocate (Fig. 3A, WF to SR). Chimeric *GFP* constructs were constructed containing either a wild type translocatable *Hoxd1* homeodomain (*d1-HD-gfp*), or a homeodomain containing a mutated penetratin sequence that blocks translocation (*mut d1-HD-gfp*) and translocation of these constructs in wraps was compared to that of wild type *GFP* as a control. Fig. 3B–D” shows that whereas wild type *GFP* and *mut d1-HD-gfp* both stay localised within the mesoderm in the wrap assay, the *d1-HD-gfp* spreads to the edges of the explant (Fig. 3C–C”). Early incubation times (0–50 min) already show spreading of the signal in an open wrap. S1 Figure also shows that the myc-tagged Hoxd1 protein travels to the edges of the recombinate, far from its initial site of expression (see S1A and B). These results clearly show that *Xenopus Hoxd1* contains a functional penetratin sequence with a cargo function as has been reported for other homeoproteins.

**Figure 4:**
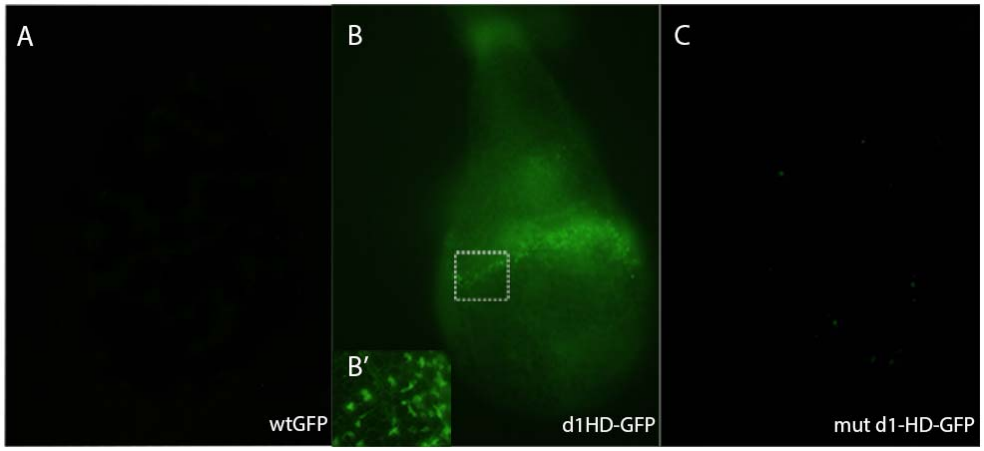
Uptake of *d1-HD-GFP* and *GFP* by *Drosophila* imaginal wing discs. *Drosphila* imaginal wing discs were incubated with *Hoxd1-HD-GFP (d1-HD-gfp)* recombinant protein (**B**) or wild type GFP protein (**A**). or mutated *mut*-*d1-HD-GFP.* Recombinant *d1-HD-gfp* was taken up by the discs while wild type GFP and *mut-d1-HD-GFP* were not. Each photo in this figure represents 10 imaginal discs giving the same result. These data clearly show the cargo function of Hoxd1 homeodomain and suggest that this uptake is by a species independent mechanism.

Another striking property of the penetratin sequence is its ability to translocate from cell to cell non species specifically. We tested whether the *Xenopus Hoxd1* homeodomain shows this property. *Drosophila* imaginal dics were incubated with wild type *GFP*, *d1-HD-gfp* or *mut d1-HD-gfp* recombinant proteins for 15 min at room temperature. Fig. 4 shows clearly that *d1-HD-gfp* (Fig. 4B, B’) is taken up by the discs while *GFP* alone (Fig. 4A) or *mut d1-HD-gfp* (Fig. 4C) do not cross the epithelial layer of the disc. This is in accordance with previous reports that showed that mutation of 2 key amino acids within the penetratin sequence abolishes the uptake of the latter [26]. This result shows a clear non species specific mechanism. This is a very robust mechanism that could allow massive information transfer between mesoderm and neurectoderm during neural transformation. This lack of species specificity is consistent with homeoprotein transfer (see discussion) but we can not rule out from the data that a highly conserved ligand-receptor mechanism (like BMP- chordin) is involved.

A phenotypic assay demonstrates functionality of the homeodomain transfer in the *Xenopus* embryo.

**Figure 5:**
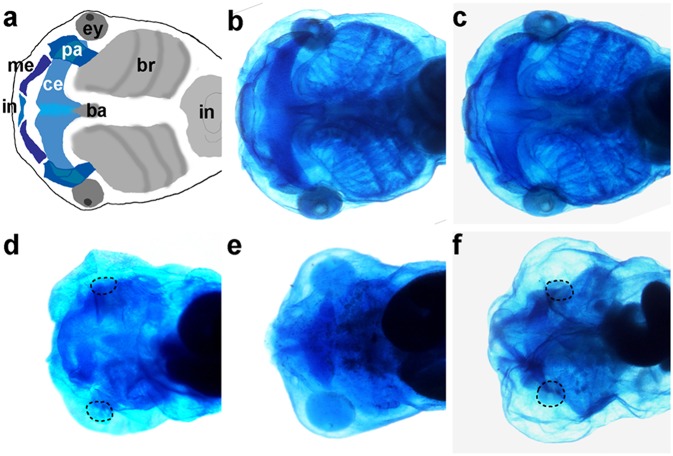
Craniofacial structures of *Xenopus laevis* tadpoles upon injection of *Hoxd1* mRNA or protein. **a**: Schematic ventral view of an uninjected untreated control embryo. **b**: uninjected embryo. **c**: embryo injected with wild type GFP into the blastoecel at blastula stage. **d**: Embryo injected with *Hoxd1* mRNA at 4 cell stage. **e**: Embryo injected with recombinant HOXD1 protein into 4 cell stage embryo. **f**: injection of recombinant HOXD1 protein into the blastoecel. Please note that standard injection of Hoxd1 mRNA or its protein counterpart injection in the cytoplasm or in the extracellular matrix leads to similar phenotypes in **d, e** and **f**. The embryos are strongly posteriorised as shown by the reduction (or deletion) of anterior structures. These data strongly suggest that the Hoxd1 protein successfully crossed the cellular membranes and retained its function as it leads to a severe truncation of anterior cartilage structures. Infrarostrale (in), Meckel’s cartilage (me), palatoquadrate (pa), ceratohyale (ce), basibranchiale (ba), branchial arches (br), eye (ey), intestine (in). Each photo represents 10 identically treated embryos giving the same result.

The ability of *Xenopus Hoxd1* homeoprotein to exhibit properties predicted by its penetratin sequence is interesting. However, this unconventional transfer was not shown to account for an embryonic function. So, we investigated whether recombinant *Hoxd1* protein exhibits biological activity when applied in the extracellular space of *Xenopus* gastrula embryos. When *Hoxd1* is overexpressed according to standard methods by injecting messenger RNA, the embryo’s craniofacial structures are strongly affected and show a severe reduction of size of the branchial arches as we have previously reported (Fig. 5d) [28]. This reflects posteriorisation of A-P positional information in the head region. A wild type recombinant *Hoxd1* protein was produced and injected either into the blastomeres of an early embryo (Fig. 5e) or into the extracellular space of the blastocoel during gastrulation (Fig. 5f). Upon injection of *Hoxd1* recombinant protein, craniofacial structures are reduced similarly as following standard mRNA injections, without any difference between the intra- and extracellular injection conditions. Conversely injection of *GFP* protein into the blastocoel during early gastrulation causes no phenotype (Fig. 5, compare b to c) excluding a possible toxic side effect due to blastoecel injections *per se*. These results show that the recombinant protein induces the same effect when applied intra- or extracellularly and demonstrate that the *Hoxd1* protein is efficiently internalized by the embryo and that it seems to retain its function.

Altogether, these results indicate a role of the conserved *Hox* transcription factors in signalling during neural transformation, and in vertical signalling.

## Discussion

Axial patterning of the central nervous system is a crucial event during development. It enables proper locomotion and behaviour of the animal. Many studies have investigated the nature of the molecules involved in neural patterning. The Spemann organizer is one major source of patterning and neuralizing molecules [1], [29]. Previous work also brought evidence that signals acting within the plane of the neural tissue (called planar signals) are necessary but not sufficient to account for the complicated A-P pattern of the neural plate [16]–[19]. Emerging evidence has also indicated the involvement of signals from the non organizer mesoderm (NOM) in several vertebrates including the frog [4]–[9]. These clearly mediate what has been called vertical signalling [2]. We have investigated the involvement of *Hox* genes in vertical signalling. *Hox* transcription factors are the key players in determining positional information along the anteroposterior axis of the vertebrate embryo. In *Xenopus laevis*, it has been shown that *Hox* gene expression is initiated in the NOM of the gastrula and then subsequently spreads to the adjacent neurectodermal cells [20]. We investigated the possibility of a link between mesodermal *Hox* expression and the subsequent *Hox* expression in the neurectoderm using a tissue recombination assay (wrap assay), that was previously proven to be an ideal way to manipulate different tissues in a controlled manner [20], [23]. We previously showed a clear requirement for a vertical, non organizer mesoderm (NOM) derived signal for the induction of neural *Hox* expression. Here, we show that the NOM *Hox* expression itself is part of the information necessary for this signal and that there is thus a clear connection between the early mesodermal and early neurectodermal *Hox* codes. Our investigations using gain and loss of function approaches revealed that this requirement for NOM *Hox* expression is specific. It is striking that the loss of function MO approach shows that each *Hox* gene we tested actually needs to be translated itself in NOM to enable its own induced expression in neurectoderm. This implies that mesodermal function of each of these Hox genes is uniquely required for its own induction in neurectoderm in the wrap recombinates used for this test. No other Hox gene or developmental regulator that is co-expressed in the recombinate can substitute this function. This is high specificity. We have so far not identified the actual signalling agents travelling between the mesoderm and neurectoderm and the possibilities exist that these are *Hox* induced downstream signal molecules or the *Hox* proteins themselves. However, it would be very difficult to account for the very high degree of specificity we see by a conventional mechanism, such as a morphogen gradient. In previous studies, the *Drosophila Hox* protein *Antennapedia* and other homeoproteins have been shown to exhibit the capacity to travel across biological membranes by means of a ‘penetratin’ sequence within the homeodomain under conditions excluding any classical endocytosis mechanism [25]–[27]. This is a much more specific mechanism and due to the extreme conservation of *Hox* proteins throughout evolution, it is likely that the presence and the conservation of sequences allowing homeoprotein internalization and secretion might be of physiological relevance during development. We thus decided to investigate if a *Xenopus Hox* protein (*Hoxd1*: the first expressed *Hox* gene) exhibits a capacity to translocate across biological membranes. We conclusively demonstrated that *Hoxd1* exhibits properties of transfer and that the transfer is active in the *Xenopus* gastrula and it does not negatively affect the functionality of the protein. This is a very specific mechanism that delivers a very specific signal, a fact that is entirely consistent with our finding that induction of the expression of a particular *Hox* gene in neurectoderm specifically requires expression of this same *Hox* gene itself (and not just, for example of one of its paralogues) in NOM mesoderm. Other studies point to unexpectedly specific actions of secreted homeoproteins in brain function [43], [44].

Gradients of *FGFs, RA* and *Wnts* have all been proposed to be involved in activation of *Hox* gene expression along the anterior-posterior axis [12]–[15]. Biologically active retinoids are good candidates for signalling from mesoderm to neurectoderm because they are synthesized in mesoderm by *RALDH* and destroyed there by *Cyp 26*, and it seems that retinoid receptors are present mainly in the neurectoderm [31]–[34]. The synthetic and degradative regions of the mesodermal axis frame the hindbrain: the axial segment where *Hox* paralogue groups *1–5* have their anterior neurectodermal boundaries and show collinearly graded *RA* sensitivities [31], [33]. In *Xenopus*, disruption of *RA* production in the mesoderm has no effect on *Hox* mesodermal expression while it only downregulates *Hox* in the neurectoderm [34]. However, *Hox* paralogue groups *6–13* do not exhibit *RA* sensitivity [31]. *FGFs* and *Cdx* might play a role in regulating neurectodermal *Hox* expression here [35], [36]. We think that these different pathways work in concert with specific *Hox* signals. In fact, it has been shown in the chordate Amphioxus that the *labial Hox* gene (*Hox1*) mediates (all of) the effects of *RA* on the hindbrain (including regulating expression of more posterior *Hox* genes) [37]. This fits the finding that knocking out the whole *Hox1* paralogue group in *Xenopus* knocks out expression of more posterior *Hox* genes as well as *Hox1* [28]. It has also been shown previously that homeoprotein transfer and conventional signalling pathways work in concert in other contexts [29], [30].

The growing evidence that homeoproteins are signalling molecules could be the key to understanding the whole picture of neural patterning. Indeed, the simplicity of *Hox* protein transfer would elegantly solve the patterning problem because it accounts for the one to one connection between mesodermal and neurectodermal *Hox* expression. *Hox* homeoprotein transfer was discovered almost 30 years ago [25], [26] but its biological relevance has been unclear until recently [38], [39], [43], [44]. We suspect that homeoprotein transfer is an ancient conserved signalling mechanism that is central to the ancestral *Hox* patterning mechanism that evolved in bilateria. Findings by Prochiantz and colleagues indicate that homeoprotein transfer is an extremely ancient form of signalling that antedates the division between animals and plants and presumably thus evolved before specific ligand-receptor mediated signalling. We suspect it has been selected and retained for vertical signalling because it is so eminently suitable to mediate *Hox* information copying.

In summary, our data show that at least several *Hox* genes expressed in the *Xenopus* gastrula show a causal connection between initial mesodermal expression and later neural expression. This connection is highly specific. The Hox protein encoded by at least one of these genes also shows unconventional intercellular transfer behaviour during vertical signalling and seems able to exert its biological function after passing through cell membranes as reported for members of other *Hox* paralogous groups in former studies.

## Summarising Conclusions

1. We show, in Figs. 1 and 2, that Hox proteins expressed in *Xenopus* gastrula NOM mesoderm induce their own expression in gastrula neurectoderm. There is thus non cell autonomous autoregulation of individual Hox genes. We equate this autoregulation with the classical concept of ‘vertical signalling’: copying of positional information from gastrula mesoderm to gastrula neurectoderm. Loss of function experiments for three different Hox genes, using morpholinos (Fig. 1), show that this homologous induction is specific for each of these three genes. Loss of function for each particular gene in NOM mesoderm deletes its own expression in neurectoderm. Clearly, no other endogenously expressed Hox gene or developmental regulator can substitute for this unique function. Fig. 2 shows that Hox expression induced in Hox gain of function exeriments is localised not only in the (SO) mesoderm in which the Hox gene was introduced into a wrap recombinate but also widely in mesoderm and in neurectoderm. See Figs. 1 and 2 for details.
2. The specificity of vertical signalling led us to consider an unorthodox mechanism. Prochiantz and colleagues have shown that Hox proteins and other homeoproteins can transfer from cell to cell. They transfer by virtue of a special ‘penetratin’ sequence within the homeobox of the homeoprotein which can be mutated to ablate transfer of the homeoprotein (internalisation or secretion). This mechanism is now well known and well accepted and has been shown to mediate several different developmental functions. It is exactly the type of mechanism needed to mediate ‘vertical signalling’, which is copying of positional information from one germ layer to another and which we have shown (above) to be mediated by copying of Hox gene expression from one germ layer to another.
3. We examined whether Prochiantz transfer occurs during vertical signalling between mesoderm and neurectoderm from *Xenopus* gastrulae. Myc tagged Hoxd1 was introduced into wrap recombinates in NOM mesoderm and cultured over 6–8 hrs. Over this time course, Myc tagged Hoxd1 spreads to every part of the recombinate, from the SO mesoderm in which it was introduced; to neurectoderm as well as mesoderm. We conclude that Hoxd1myc is transferred from cell to cell. (See S1 Figure). We also tested the dependence of this transfer on ‘penetratin’. We thus constructed a chimeric protein consisting of Hoxd1 homeodomain (including the penetratin sequence) coupled to *GFP*. We tested the mobility of this labelled protein in comparison to a mutant where two essential amino acids in the penetratin sequence are mutated and the capacity for transfer is ablated; and with GFP itself. The wild type Hoxd1HD-GFP (*d1-HD-gfp*) was transferred from mesoderm to neurectoderm (transfer made more visible using red phalloidin background staining). The mutated homeodomain coupled to *GFP* protein (*mut d1-HD-gfp*) and GFP were not. We conclude that the homeodomain of the Hox gene Hoxd1 transfers from mesoderm to neurectoderm (Fig. 3). And that Hoxd1 itself is indeed transferred from gastrula mesoderm to gastrula neurectoderm by the Prochiantz mechanism ( S1 Figure).
4. We tested the species specificity of *d1-HD-gfp* homeoprotein transfer. This was taken up by Drosophila imaginal discs whereas GFP and *mut-d1-HD-GFP* were not. This finding is consistent with the known extreme aspecificity of Prochiantz signalling (which occurs both in animals and plants and presumably evolved very early on, possibly before ligand-receptor signalling). This emphasizes that Hoxd1 passes cell membranes in a species independent manner, confirming the previous indications of species independent Hox transfer and excluding a requirement for species specific forms of ligand-receptor signalling (Fig. 4).
5. We tested the functionality of Hoxd1 protein transfer. When this protein was injected into the blastocoel of *Xenopus* gastrulae, it gave the same posteriorising phenotype as cytoplasmically injected Hoxd1 mRNA or protein. This again emphasizes that Hoxd1 protein passes cell membranes and that the transported protein is functional.
6. Finally, the findings above demonstrate unambiguously that non cell autonomous autoregulation of Hox proteins mediates vertical signalling/neural transformation in the *Xenopus* gastrula and that at least for Hoxd1, a mechanism involved is Prochiantz intercellular homeoprotein transfer.

## Methods Summary

### Ethics

The Leiden University Animal Experimentation Ethics (DEC) Committee have approved this work.

### Handling and treating *Xenopus* embryos

Embryos were staged according to Nieuwkoop and Faber [40]. *In vitro* fertilization, embryo culture, mRNA and antisense morpholino oligonucleotides (MO, Gene-Tools Inc.) and protein injections were used as previously described [41]. *In situ* hybridization analyses were carried out as formerly reported [41]. The wrap assay was as described previously [41]. Microsurgery was carried out using hair knives. Mesodermal tissue (NOM or SO) was explanted and the epithelial layer removed. After keeping these tissue explants for a few minutes in MBS, they were placed between two animal caps, which had been cut immediately before to prevent curling. Wraps were cultivated in 1%MBS for about 30 min and then transferred to 0.1% MBS. Wraps were usually fixed 6 to 8 h after preparing them, when control embryos reached stage 14–15 (i.e. mid neurula).

### Preparation of recombinant GFP proteins

Preparation of recombinant *GFP* poteins were as described [42]. Primers for the synthesis of cargo proteins *Hoxd1* homeodomain (wild type and mutant) linked to *GFP* are as follows: Forward – GGGATCCATGCCCTGCAATGTGAGGACAAA; and Reverse – GGAATTCATTCCACCCCCTCCGCCACCCCTTTCCCTTTTCTTTTGTTTCA for the wild type *Hoxd1* homeodomain (*d1-HD-gfp*); and for the mutated *Hoxd1* homeodomain (*mut d1-HD-g*fp), the primers are: Forward - GAAGATCTCTCGACAGAACAGAAGAATGAAACAAAA; and Reverse - CCCAAGCTTCTAGGGTGAAGCGTCCTTGGA. MO’s sequences are: *Hoxd*1 MO [28]; *Hoxb9* MO: 5′ – TGACCGACCCACCAGCTTCTCCCCA −3′; and the standard control morpholino (ctMO) from Genes-Tools Inc.

### Immunolocalisation

Immunolocalisation was performed using monoclonal anti-*myc* tag antibody (Sigma-Aldrich), and a secondary anti-mouse Alexa-488 antibody (Invitrogen) according to a previously described protocol [34]. Wraps were made as described earlier [23]. Wraps were bisected prior to cryosectioning (5 µm) and processed for *in situ* or immunolocalisation, and *in situ* was performed before being embedded in gelatin and sectioned (10 µm).

### Phalloidin counterstaining

Wraps were bisected prior to phalloidin (Invitrogen) step because the uptake by the cells was greatly improved.

### *Drosophila* Imaginal Discs

*Drosophila* larvae were dissected in PBS. Imaginal discs were incubated in PBS + recombinant proteins at room temperature for 15 min. The discs were then extensively washed with PBS prior to analysis. Each experiment is based on 20 or more discs.

### Numbers

In each of the experiments in Figs. 1–5, S1 and S2 Figures, each photo is representative of 20 to more recombinates or embryos or imaginal discs, all of which gave the same result. See figure legends for details.

## Supporting Information

S1 Figure **Immunolocalisation of Hoxd1-myc in wraps.** Immunolocalisation of *Myc-* labelled *Hoxd1* protein after 6–8 hrs of culture in a wrap. **A**: control wrap. **B**: immunolocalisation of myc-tagged Hoxd1 in a wrap containing [AC(SO+*d1-myc*)+SO)AC] shows spreading of *myc*-Hoxd1 protein from mesoderm to the outer layers of the wrap (neurectoderm) while no signal is detected in the control wrap (**A**). The signal spreads throughout the recombinate to originally unlabelled neurectoderm as well as originally unlabelled mesoderm. Each photo represents 10 wraps giving identical results.
(TIF)

S2 Figure **Open wrap reveals an early spreading of the cargo d1-HD-GFP.** **A**: Left, a wrap containing [AC(SO+*mut-d1-HD-gfp*)SO)AC]. The upper animal cap is replaced by a small glass coated with BSA. This allows us to follow changes of the signal in time. Right, a wrap containing [AC(*SO+d1-HD-gfp*)SO)AC]. **B**: after 30 min of incubation, both wraps stayed open. **C**: after 50 min of incubation, the signal in the wrap with d1-HD-gfp shows a little spreading towards the outside of the SO. This shows that, even after such a short incubation time, the penetratin is capable of playing its cargo function for the GFP protein. Each photo represents 10 wraps giving the same result.
(TIF)
