# Supplementary figures and images for: Vertical Signalling Involves Transmission of Hox Information from Gastrula Mesoderm to Neurectoderm
Source: PLoS One. 2014 Dec 16;9(12):e115208. doi: 10.1371/journal.pone.0115208 (PMC4267835; doi:10.1371/journal.pone.0115208)

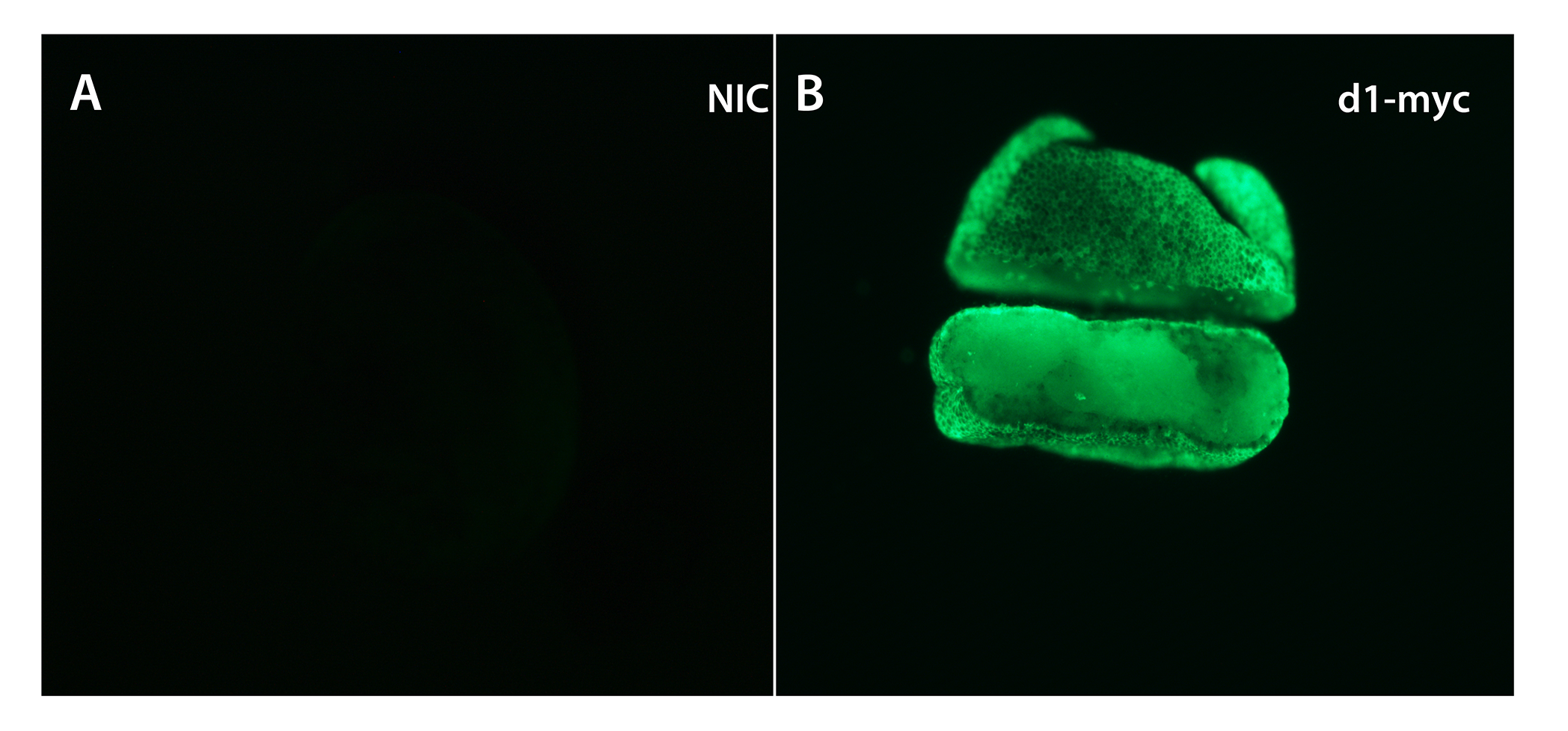

Supplement: S1 Figure — Immunolocalisation of Hoxd1-myc in wraps. Immunolocalisation of Myc- labelled Hoxd1 protein after 6–8 hrs of culture in a wrap. A: control wrap. B: immunolocalisation of myc-tagged Hoxd1 in a wrap containing [AC(SO+d1-myc)+SO)AC] shows spreading of myc-Hoxd1 protein from mesoderm to the outer layers of the wrap (neurectoderm) while no signal is detected in the control wrap (A). The signal spreads throughout the recombinate to originally unlabelled neurectoderm as well as originally unlabelled mesoderm. Each photo represents 10 wraps giving identical results. (TIF) [file pone.0115208.s001.tif]

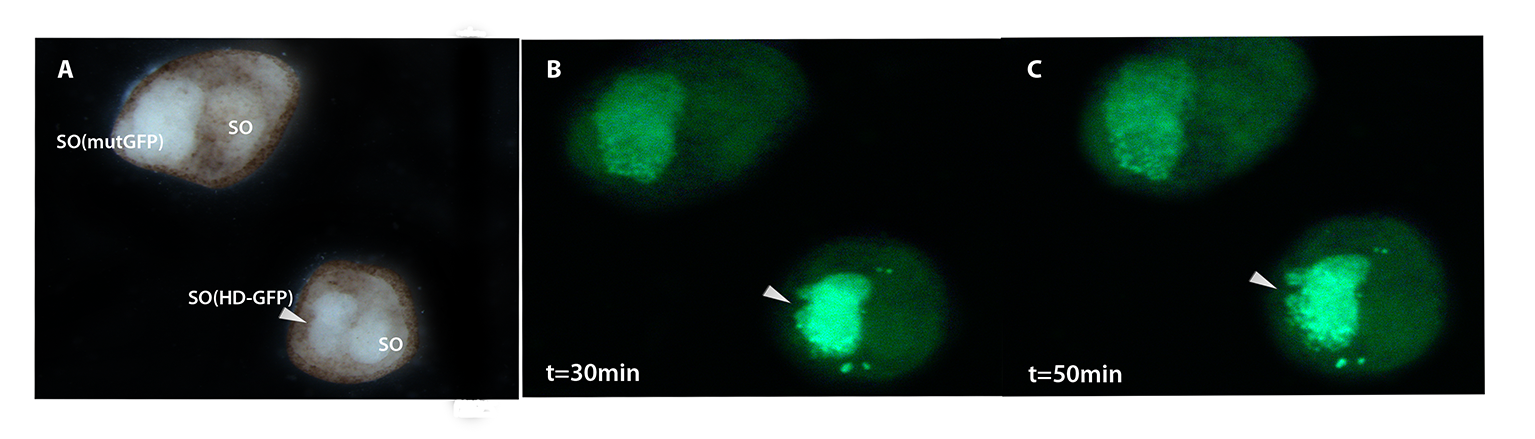

Supplement: S2 Figure — Open wrap reveals an early spreading of the cargo d1-HD-GFP. A: Left, a wrap containing [AC(SO+mut-d1-HD-gfp)SO)AC]. The upper animal cap is replaced by a small glass coated with BSA. This allows us to follow changes of the signal in time. Right, a wrap containing [AC(SO+d1-HD-gfp)SO)AC]. B: after 30 min of incubation, both wraps stayed open. C: after 50 min of incubation, the signal in the wrap with d1-HD-gfp shows a little spreading towards the outside of the SO. This shows that, even after such a short incubation time, the penetratin is capable of playing its cargo function for the GFP protein. Each photo represents 10 wraps giving the same result. (TIF) [file pone.0115208.s002.tif]
